# Identification and Antioxidant Properties of Phenolic Compounds during Production of Bread from Purple Wheat Grains

**DOI:** 10.3390/molecules200915525

**Published:** 2015-08-26

**Authors:** Lilei Yu, Trust Beta

**Affiliations:** 1 Department of Food Science, University of Manitoba, Winnipeg, MB R3T 2N2, Canada; E-Mail: umyul@myumanitoba.ca; 2 Richardson Centre for Functional Foods & Nutraceuticals, University of Manitoba, Smartpark, Winnipeg, MB R3T 2N2, Canada

**Keywords:** purple wheat, bread-making, phenolic acids, anthocyanins, antioxidant activity, HPLC analysis

## Abstract

Phenolic profiles and antioxidant properties of purple wheat varieties were investigated to document the effects of bread-making. Bread crust and crumb along with samples collected after mixing, 30 min fermenting, 65 min fermenting, and baking were examined. Free phenolic content (105.4 to 113.2 mg FAE/100 g) significantly (*p* < 0.05) increased during mixing, fermenting, and baking (65% to 68%). Bound phenolics slightly (*p* > 0.05) decreased after 30 min fermentation (7% to 9%) compared to the dough after mixing, but increased significantly (*p* < 0.05) during 65 min fermenting and baking (16% to 27%). Their antioxidant activities followed a similar trend as observed for total phenolic content. The bread crust demonstrated increased free (103% to 109%) but decreased bound (2% to 3%) phenolic content, whereas bread crumb exhibited a reversal of these results. Total anthocyanin content (TAC) significantly (*p* < 0.05) decreased by 21% after mixing; however, it gradually increased to 90% of the original levels after fermenting. Baking significantly (*p* < 0.05) decreased TAC by 55%, resulting in the lowest value for bread crust (0.8 to 4.4 mg cyn-3-glu equiv./100 g). *p-*Hydroxybenzoic, vanillic, *p-*coumaric, and ferulic acids were detected in free-phenolic extracts, while protocatechuic, caffeic syringic, and sinapic were additional acids in bound-phenolic extracts. Cyanidin-3-glucoside was the detectable anthocyanin in purple wheat. Bread-making significantly (*p* < 0.05) increased the phenolic content and antioxidant activities; however, it compromised the anthocyanin content of purple wheat bread.

## 1. Introduction

Wheat, as one of the staple foods worldwide, is not only regarded a source of protein and carbohydrates, but is also recognized for its potential in reducing the risk of oxidative-stress related chronic diseases and age-related disorders, such as cardiovascular diseases, neurodegeneration, type II diabetes, obesity, and some cancers [1,2]. The potential health benefits are partly attributed to its unique phytochemical composition. In recent years, these trace amounts of antioxidant phytochemicals have attracted considerable interest from both researchers and food manufacturers. Phenolic compounds are present in wheat as secondary plant metabolites for normal functions [3]. The most abundant antioxidants present in wheat are phenolic acids [4]. The substances that contribute to the distinction of pigmented wheat and common wheat are anthocyanins. Anthocyanins contribute significantly to the antioxidant activity of colored wheat [5].

Generally whole wheat, as harvested, is not ingested directly by humans, but requires some processing prior to consumption. Bread, as one of the processed cereals made from wheat, is an important food product in human diets and therefore acts as a suitable carrier for health-promoting compounds. There is growing consumer interest in ingesting whole-meal products because of the concentrated dietary fiber and phytochemicals in the outer layers of the grains [6,7]. Even though increasing evidence has indicated the health potential of wheat, food manufacturing may compromise its functional properties, resulting in loss of antioxidant activity. There is substantial literature on the *in vitro* antioxidant properties of phenolic acids and anthocyanins. However, their specific changes, at each critical step of the bread-making process, have not been adequately investigated. Therefore, the present research aimed to investigate the changes of phenolic acids and anthocyanins as well as their antioxidant activities during bread production.

## 2. Results and Discussion

### 2.1. Free, Bound, and Total Phenolic Content Using the Folin-Ciocalteau Method

Table 1 summarizes the amount of soluble free, insoluble bound, and total phenolic content at different stages of the bread-making process. Results were expressed as mg ferulic acid equivalent (FAE) and gallic acid equivalent (GAE)/100 g. Ferulic acid (FA) was used as a standard because of its predominance among phenolic acids in wheat. Even though gallic acid (GA) was rarely detected in wheat, it was still used for a comprehensive comparison among the literature.

**Table 1:** Free, bound, and total phenolic content of common and purple wheat grains at different stages of the bread-making process.

Ferulic Acid Equivalent									
	Free Phenolic Content (mg FAE/100 g)			Bound Phenolic Content (mg FAE/100 g)			Total Phenolic Content (mg FAE/100 g)		
	Öelands hvede ^A^	Indigo ^A^	Konini ^A^	Öelands hvede ^B^	Indigo ^B^	Konini ^B^	Öelands hvede	Indigo	Konini
Flour	113.23 ± 1.97 ^kl^	105.44 ± 0.82 ^kl^	111.26 ± 0.49 ^kl^	125.36 ± 5.26 ^efg^	121.82 ± 3.95 ^efg^	123.68 ± 5.53 ^efg^	238.59 ± 7.24	227.26 ± 4.77	234.94 ± 6.02
Mixing	129.05 ± 1.64 ^ghi^	125.76 ± 4.93 ^ij^	128.70 ± 0.82 ^ghi^	138.57 ± 2.37 ^bc^	135.96 ± 2.37 ^bcd^	137.08 ± 1.32 ^bcd^	267.62 ± 4.01	261.72 ± 7.30	265.78 ± 2.14
30 min fermenting	136.14 ± 4.77 ^efg^	128 ± 2.47 ^hi^	135.67 ± 1.48 ^efgh^	129.64 ± 2.37 ^cde^	125.73 ± 5.79 ^efg^	127.96 ± 2.63 ^def^	265.78 ± 7.14	253.73 ± 8.26	263.63 ± 4.11
65min fermenting	142.3 ± 0.66 ^e^	130.21 ± 4.28 ^fghi^	137.53 ± 6.74 ^ef^	141.73 ± 5.26 ^b^	138.20 ± 5.53 ^bc^	140.61 ± 4.74 ^b^	284.03 ± 5.92	268.41 ± 9.80	278.14 ± 11.48
Bread loaf	185.91 ± 3.78 ^c^	176.72 ± 0.99 ^d^	183.53 ± 2.96 ^cd^	158.10 ± 5.79 ^a^	154.75 ± 5.79 ^a^	156.43 ± 3.95 ^a^	344.01 ± 9.57	331.47 ± 6.78	339.96 ± 6.91
Bread crust	228.81 ± 5.92 ^a^	219.16 ± 6.08 ^b^	229.86 ± 1.81 ^a^	122.57 ± 5.00 ^efg^	116.80 ± 3.16 ^g^	119.59 ± 6.05 ^gf^	351.38 ± 10.92	335.96 ± 9.24	349.45 ± 7.86
Bread crumb	123.81 ± 6.08 ^ij^	114.28 ± 3.78 ^k^	119.05 ± 5.26 ^jk^	163.13 ± 0.26 ^a^	159.22 ± 4.74 ^a^	161.64 ± 8.16 ^a^	286.94 ± 6.35	273.50 ± 8.52	280.69 ± 13.42
**Gallic Acid Equivalent**									
	**Free Phenolic Content (mg GAE/100 g)**			**Bound Phenolic Content (mg GAE/100 g)**			**Total Phenolic Content (mg GAE/100 g)**		
	**Öelands hvede ^C^**	**Indigo ^C^**	**Konini ^C^**	**Öelands hvede ^D^**	**Indigo ^D^**	**Konini ^D^**	**Öelands hvede**	**Indigo**	**Konini**
Flour	83.15 ± 1.37 ^kl^	77.74 ± 0.57 ^kl^	81.77 ± 0.34 ^kl^	94.32 ± 3.65 ^efg^	91.87 ± 2.74 ^efg^	93.16 ± 3.83 ^efg^	177.47 ± 5.02	169.61 ± 3.31	174.94 ± 4.17
Mixing	94.11 ± 1.14 ^ghi^	91.85 ± 1.14 ^ij^	93.87 ± 0.57 ^efgh^	103.48 ± 1.64 ^bc^	101.68 ± 1.64 ^bcd^	102.45 ± 0.91 ^bcd^	197.60 ± 2.78	193.53 ± 5.06	196.32 ± 1.48
30 min fermenting	99.03 ± 3.31 ^efg^	93.39 ± 1.71 ^hi^	98.71 ± 1.03 ^efgh^	97.29 ± 1.64 ^cde^	94.58 ± 4.01 ^efg^	96.13 ± 1.82 ^def^	196.32 ± 4.95	187.97 ± 5.73	194.84 ± 2.85
65 min fermenting	103.31 ± 0.46 ^e^	94.92 ± 0.46 ^fghi^	100.00 ± 4.68 ^ef^	105.68 ± 3.65 ^b^	103.23 ± 3.83 ^bc^	104.90 ± 3.28 ^b^	208.98 ± 4.11	198.15 ± 6.80	204.90 ± 7.96
Bread loaf	133.55 ± 2.62 ^c^	127.18 ± 2.62 ^d^	131.21 ± 2.05 ^cd^	117.03 ± 4.01 ^a^	114.71 ±4.01 ^a^	115.87 ± 2.74 ^a^	250.58 ± 6.64	241.89 ± 4.70	247.08 ± 4.79
Bread crust	163.31 ± 4.11 ^a^	156.61 ± 4.11 ^b^	164.03 ± 1.25 ^a^	92.39 ± 3.47 ^efg^	88.39 ± 2.19 ^g^	90.32 ± 4.20 ^fg^	255.69 ± 7.57	245.00 ± 6.41	254.35 ± 5.45
Bread crumb	90.48 ± 4.22 ^ij^	83.87 ± 4.22 ^k^	87.18 ± 3.65 ^jk^	120.52 ± 0.18 ^a^	117.81 ± 3.28 ^a^	119.48 ± 5.66 ^a^	211.00 ± 4.40	201.68 ± 5.91	206.66 ± 9.31

^A–D^ Columns labeled with the same capital superscript were considered as a group; ^a–^^l^ Significant difference was defined with different letters in each group. Total phenolic content was calculated as the sum of free and bound phenolic content for each raw.

As seen in Table 1, the free phenolic content (FPC) of raw wheat flour varied between 113.2, 105.4, and 111.3 mg FAE/100 g for Öelands hvede, Indigo, and Konini, respectively. The levels were slightly higher than the 100% methanol extracts of black wheat (70.6 to 110.8 mg FAE/100 g) reported by Li *et al.* [8], but lower than the acidified methanol extracts of colored wheat (146 to 226 mg FAE/100 g) reported by Liu *et al.* [5]. Dough mixing significantly (*p* < 0.05) increased FPC by 14.0% to 19.3%. Fermentation also remarkably increased FPC, but its effect was not significant (*p* > 0.05) during the first 30 min. FPC significantly (*p* < 0.05) increased during baking by 64.2% to 67.6%. Bread crust contained the highest FPC, while bread crumb contained a relatively lower FPC. Among the three wheat varieties, Öelands hvede had the highest FPC, whereas the purple wheat, Indigo, contained the lowest value. This difference was not significant (*p* > 0.05) until the process of 30 min fermentation.

The bound phenolic content (BPC) of raw flour varied between 94.3, 91.9, and 93.2 GAE/100 g for Öelands hvede, Indigo, and Konini, respectively. The levels were in agreement with those reported by Adom *et al.* [9], who investigated BPC in whole wheat varieties with a range of 86 to 128 mg GAE/100 g, slightly lower than those (93 to 113 mg GAE/100 g) reported by Okarter *et al.* [2]. Significant (*p* < 0.05) increase in BPC occurred during mixing (10.5%–11.6%) and baking (26.1%–27.0%); however, BPC decreased to almost the same level as raw flour after 30 min fermentation. During the next 35 min (65 min fermentation), BPC significantly (*p* < 0.05) increased by 13.1%–13.7% compared to raw flour. Unlike FPC, the bread crumb had the highest BPC, while the bread crust contained the lowest value. No significant difference (*p* > 0.05) was observed among the three wheat varieties throughout the bread-making process.

The major increase in total phenolic content (TPC) was obtained after mixing and baking. TPC gradually increased during dough fermentation and the major increase occurred at the last 35 min fermenting step. Bread crust contained the highest TPC, followed by whole bread and bread crumb. These results were in accordance with the findings investigated by Perez-Jimenez *et al.* [10], who concluded that bread and bread crust had significantly higher content (Folin–Ciocalteu method) of non-extractable antioxidants than wheat flour. Gelinas and McKinnon [11] also reported that baking increased the concentration of phenolic compounds while the bread crust contained more phenolic compounds than the crumb.

The functions of intrinsic phenolic antioxidants as structural components in wheat grains have been fully described in the literature. For example, ferulic acid is found to be ester-bound to arabinoxylans [12,13] or ether-linked to lignin or lignin-like polymers [14]. Phenolic compounds also form complexes with proteins via hydrogen, covalent, or ionic bonds, and hydrophobic interactions [15,16,17,18]. During mixing, some intrinsic enzymes might be activated upon the addition of water. Along with the mechanical shearing effect, the phenolic complexes might be partially broken down. The increase in FPC was most likely due to the release of some bound phenolic compounds into their free forms. Continuous release of bound phenolic compounds was evidenced by the increase in FPC but decrease in BPC during the first 30 min fermentation. Baking significantly (*p* < 0.05) increased the levels of FPC and BPC by approximately 65% and 26%, respectively. This was most likely due to the complex mechanism of the baking process, which involved starch gelatinization/pasting, protein denaturation, and a Maillard reaction [18]. These changes occurred simultaneously and possibly led to the release of bound phenolic compounds. Furthermore, studies showed that the Maillard Reaction Products (MRPs) with reductone-type structure or phenol-like complexes exhibited antioxidant activity and were expected to interfere with the absorbance of Folin–Ciocalteu assay [19,20]. The use of 80% methanol largely incorporated aqueous MRPs into the extract, therefore most likely being detectable in the bread extract. Besides amino acids and reducing sugar, a recent study found that MRPs also formed from phenolic compounds, including phenolic acids and flavonoids [10]. For example, ferulic acid was incorporated into the melanoidins, contributing significantly to the higher content of bread TPC using Folin assay [10].

In terms of bread fractions, bread crust contained the highest FPC, followed by whole loaf. Bread crumb contained the lowest value. Nevertheless, BPC was better recovered in bread crumb than in crust. This was most likely due to the concentrated MRPs in bread crust. Borrelli *et al.* [21] indicated that the lower temperature but higher water activity inside the dough caused the light color of bread crumb leading to fewer MRPs in bread crumb.

### 2.2. Antioxidant Activities (AOAs) of Soluble and Insoluble Phenolic Compounds

**Figure 1:**
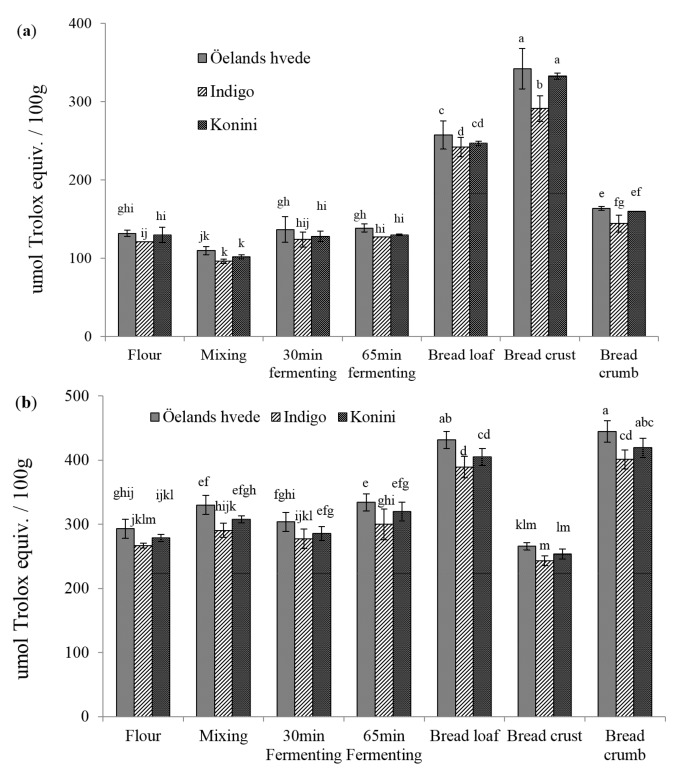
DPPH radical scavenging capacity of soluble (**a**) and insoluble (**b**) phenolic compounds. Columns marked by different letters are significantly different (*p* < 0.05).

The DPPH• scavenging activities of soluble phenolic extracts are shown in Figure 1a. The values varied between 139, 127, to 130 μmol trolox equivalent (TE)/100 g for Öelands hvede, Indigo, and Konini, respectively. The DPPH levels for Öelands hvede, Indigo, and Konini significantly (*p* < 0.05) decreased by 17%, 21%, and 22%, respectively, after mixing. This could be explained by the dilution of raw flour with non-antioxidant bread ingredients. The DPPH values recovered to the initial levels during the first 30 min fermentation, and slightly increased in the next 35 min. Baking significantly (*p* < 0.05) increased DPPH• scavenging activity by 85%, 91%, and 90% for Öelands hvede, Indigo, and Konini, respectively. The increasing effect was better seen in bread crust than in crumb. Elevated AOAs of soluble phenolic extracts were also reported by Yu *et al.* [22] for bread samples. The MRPs-melanoidins have been considered to contribute significantly to the antioxidant properties of baked grain products [23]. No significant (*p* > 0.05) difference among wheat varieties was detected in raw flours during mixing and fermenting. However, a significantly (*p* < 0.05) lower DPPH level of Indigo was detected after baking.

Figure 1b shows the DPPH values for insoluble phenolic compounds. The levels for raw flour were double those of soluble phenolic extracts. On the contrary to soluble phenolic extract, mixing significantly (*p* < 0.05) increased the DPPH• scavenging activity except for Indigo flour (increased by 13%, 9%, and 10% for Öelands hvede, Indigo, and Konini). The elevated levels of BPC detected during mixing could contribute to the increase in AOAs of insoluble phenolic extracts. Also, the insoluble phenolic compounds might be less likely to undergo oxidation than soluble phenolic compounds. During fermentation, a slight decrease in DPPH values occurred in the first 30 min and then increased (*p* > 0.05) during the next 35 min. Baking significantly (*p* < 0.05) elevated the DPPH levels by 47%, 46%, and 45% for Öelands hvede, Indigo, and Konini, respectively. Unlike the DPPH• scavenging activity of soluble phenolic extract, that of insoluble phenolic compounds was considerably higher in bread crumb than in crust. This finding was consistent with BPC in bread, where crust contained the lowest while crumb had the highest value. The lower AOA of bread crust could be attributed to the loss of some phenolic compounds, which were destroyed at high temperature [24].

**Figure 2:**
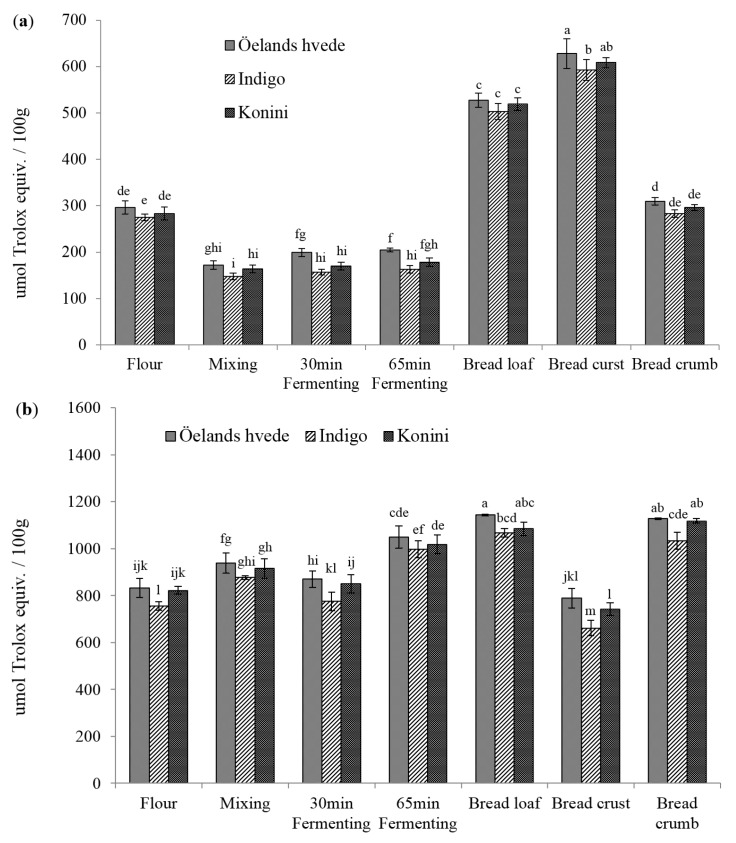
ABTS radical cation decolorization activity of soluble (**a**) and insoluble (**b**) phenolic compounds extract. Columns marked by different letters are significantly different (*p* < 0.05).

The ABTS•^+^ scavenging capacity of soluble phenolic extract in raw flour varied between 296, 275, and 283 μmol TE/100g for Öelands hvede, Indigo, and Konini, respectively (Figure 2a). A lower range of 201 to 248 μmol TE/100 g for non-colored whole wheat flour was observed by Lv *et al.* [25] using 50% acetone. Consistent to DPPH assay, ABTS method detected a significant (*p* < 0.05) decrease in AOA during mixing (decreased by 42%–46%) and a slight (*p* > 0.05) increase during fermentation. Baking significantly (*p* < 0.05) increased the AOA levels by 78% to 83% compared to raw flour. Bread crust contained the highest ABTS value (628, 592, and 608 μmol TE/100 g for Öelands hvede, Indigo, and Konini, respectively), followed by whole bread and bread crumb.

The ABTS values of insoluble bound phenolic extract in raw flour varied between 832, 755, and 822 μmol TE/100 g for Öelands hvede, Indigo, and Konini, respectively (Figure 2b). Similar to the findings with DPPH assay, the ABTS values were significantly (*p* < 0.05) higher after mixing (increased by 11%–16%). Even though a significant (*p* < 0.05) decrease in AOA occurred during the first 30 min of fermentation (decreased 7%–11% compared to mixing dough), the ABTS value increased again during the next 35 min. Baking significantly (*p* < 0.05) increased the ABTS value by 32% to 41% compared to raw flour. Bread crumb contained the highest AOA while bread crust possessed the lowest value. Compared to DPPH assay, significant difference was detected during mixing and fermenting using the ABTS method. The higher sensibility might be partially due to the solubility and diffusivity of free radicals in organic solvent and/or due to the reaction ability of native substitutes in extracts, such as phenolic acids, flavonoids, and MRPs, to DPPH and ABTS radicals.

### 2.3. Identification and Quantification of Phenolic Acids in Soluble and Insoluble Phenolic Extracts

**Figure 3:**
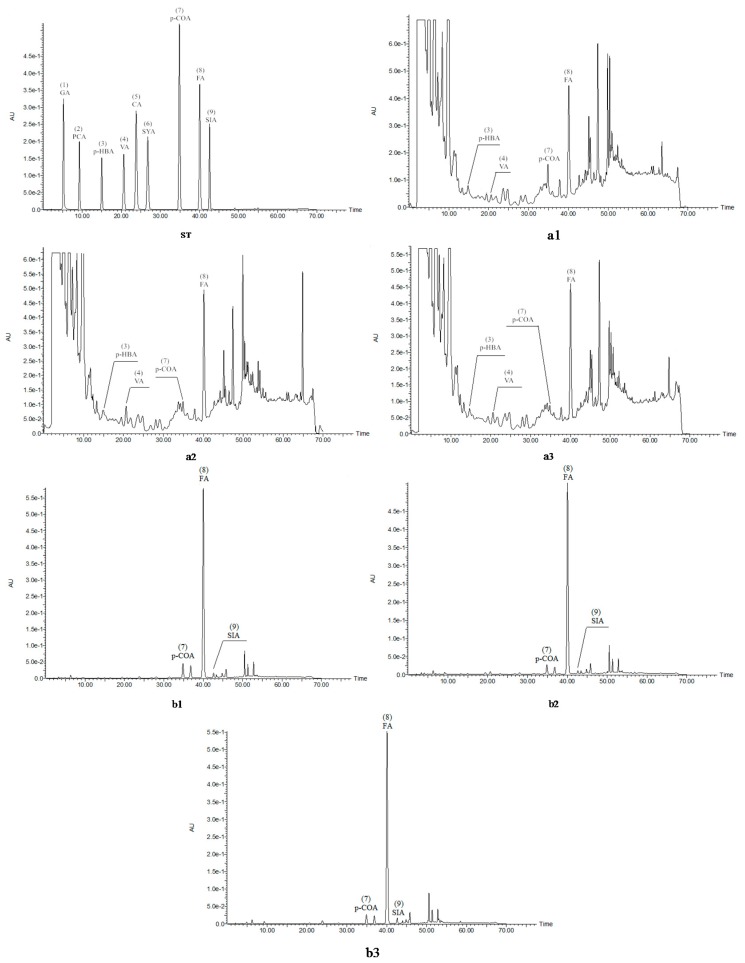
HPLC chromatograms of soluble-free (**a**) and insoluble-bound phenolic extracts (**b**) from Öelands hvede (**1**), Indigo (**2**), and Konini (**3**) bread samples at 280 nm. ST, phenolic acid standards: (**1**) gallic acid, (**2**) protocatechuic acid, (**3**) *p-*hydroxybenzoic acid, (**4**) vanillic acid, (**5**) caffeic acid, (**6**) syringic acid, (**7**) *p-*coumaric acid, (**8**) ferulic acid, and (**9**) sinapic acid.

Monomeric phenolic acids in soluble-free and insoluble-bound phenolic extracts were identified using HPLC. Identification and quantification were performed at a wavelength of 280 nm, which was confirmed to be more capable for detecting all phenolic acids in previous studies by Guo and Beta [26] and Qui *et al.* [27]. Figure 3 summarizes the chromatograms of soluble-free and insoluble-bound phenolic extracts of bread made from the three wheat varieties. The detected monomeric phenolic acids were labeled and numbered according to the retention time. In soluble-free phenolic extracts, *p-*hydroxybenzoic, vanillic, *p-*coumaric, and ferulic acid were detected; other than gallic acid, eight phenolic acids were identified in alkaline extracts.

Table 2 shows the content of detected monomeric phenolic acids in soluble fraction during bread production. In raw wheat flour, only free ferulic acid was detected with values of 2.5, 2.0, and 2.3 μg/g for Öelands hvede, Indigo, and Konini, respectively. This was in agreement with those examined by Zhang *et al.* (2 to 8 μg/g) [28] and Whent *et al.* (1.88 to 1.91 μg/g) [29]. Upon mixing flour formulation, the content of free ferulic acid significantly (*p <* 0.05) increased, up to five times the initial level. During fermentation, free ferulic acid kept increasing; meanwhile, some other free phenolic acids such as *p-*hydroxybenzoic acid, vanillic acid, and *p-*coumaric acid were detected. This phenomenon confirmed the hypothesis that mixing and fermenting facilitated the release of bound phenolic compounds into free forms. *p-*Hydroxybenzoic, vanillic, *p-*coumaric, and ferulic acid were all detected in the bread of three wheat varieties, indicating the remarkable effect of baking. Angioloni and Collar [30] also indicated that some phenolic acids, such as protocatechuic, sinapic, syringic, and ferulic acid were detected after bread-making but not in the raw flour. Free ferulic acid increased throughout the process of mixing, proofing, and baking. This finding agreed with the study conducted by Hansen *et al.* [31], who examined free ferulic acid in rye wholemeal. In bread samples, ferulic acid was predominant, followed by *p-*hydroxybenzoic, vanillic, and *p-*coumaric acid making up 45% to 51%, 26% to 37%, 11% to 18%, and 4% to 5% of total phenolic acid content, respectively. Higher contents of ferulic and *p-*hydroxybenzoic acids were found in bread crumb than in crust. This suggested that some free phenolic acids were thermally labile (high intense heat in bread crust). With respect to vanillic and *p-*coumaric acid, the levels were too low to make any conclusions.

Table 3 summarizes the contents of detected monomeric phenolic acids in insoluble fraction during the production of bread. Data on phenolic composition at each stage of bread-making in purple wheat were not previously available in the literature. Ferulic acid accounted for 77% to 82%, 78% to 83%, and 77% to 83% of total bound phenolic acid in Öelands hvede, Indigo, and Konini, respectively, throughout the process of bread-making. This result was in accordance with previous studies on the phenolic profile of whole wheat [2,5,28]. Moderate levels of phenolic acids were found as sinapic and *p-*coumaric acid, making up approximately 2.4% to 3.9% and 6.3% to 10.5% of total phenolic acids. The least abundant phenolic acids were protocatechuic, *p-*hydroxybenzoic, vanillic, caffeic, and syringic acid, having content lower than 20 μg/g.

In terms of the bread-making process, mixing increased the content of seven phenolic acids, except for caffeic acid. The first 30 min fermenting step led to a significant (*p* < 0.05) decrease in phenolic acids (only vanillic acid decreased slightly, *p* > 0.05). Total phenolic acid decreased by 26% to 27% compared to the dough after mixing. The decrease was mainly attributed to the loss of ferulic acid. This phenomenon provided evidence for the release of bound phenolic acids into their free forms. However, when the dough was fermented for 65 min, the content of total phenolic acid conversely increased to almost the same level as the mixing dough. The prolonged fermentation step helped liberate the bond between phenolic acids and insoluble large compounds, such as dietary fiber, thus making it easier to extract the “freed” phenolics. Baking significantly (*p* < 0.05) increased the content of phenolic acids, increasing up to 15% of total phenolic acid compared to the raw flour. Yu *et al.* [22] also observed a significant increase of bound ferulic acid after baking.

With regards to individual phenolic acids, the content of protocatechuic and *p-*hydroxybenzoic acid became lower after baking. In bread crust, the content of protocatechuic acid was outside the detection limit, while *p-*hydroxybenzoic acid significantly (*p* < 0.05) decreased. Higher levels of these two acids were found in bread crumb because of their indirect exposure to heat. This suggested that they are heat sensitive during the thermal process. Similar conclusions could be made about vanillic and syringic acid, whose contents increased during baking but were detected in lower levels in bread crust. Most likely, these types of phenolic acids were still released through heating but were labile to intense heat. Therefore, at a moderate level of heat, the content tended to slightly increase. However, when high heat was applied, they would degrade to their derivatives. Caffeic, *p-*coumaric, ferulic, and sinapic acid increased during baking and the contents were even higher in bread crust than in crumb. This result was consistent with the findings of Moore *et al.* [32], who reported that higher levels of insoluble bound ferulic acid were better recovered in baked pizza crust. The different behaviors of individual phenolic acids might be ascribed to their difference in chemical nature, sensitivity towards heat, and ease of liberation from the plant cell wall.

With respect to total phenolic acid content, the upper crust fraction contained the highest quantities of phenolic acids among other fractions (bread loaf and crumb). By comparison with free phenolic acid, the content of bound phenolic acid accounted for 97% to 99% of total phenolic acid content. This finding was in agreement with Zhang *et al.* [28], who investigated 37 Chinese winter wheat cultivars, concluding that free phenolic acids made up 2.5% of total phenolic content.

**Table 2:** Composition of soluble-free phenolic acids in normal and purple wheat grains at different stages of the bread-making process.

Steps	Sample Name	*p-*Hydroxybenzoic Acid	Vanillic Acid	*p-*Coumaric Acid	Ferulic Acid	Total Phenolic Acid
Flour	Öelands hvede	nd	nd	nd	2.50 ± 0.20 ^j^	2.50 ± 0.20
	Indigo	nd	nd	nd	2.02 ± 0.07 ^j^	2.02 ± 0.07
	Konini	nd	nd	nd	2.25 ± 0.01 ^j^	2.25 ± 0.01
Mixing	Öelands hvede	nd	nd	nd	10.01 ± 1.23 ^hi^	10.01 ± 1.23
	Indigo	nd	nd	nd	9.04 ± 0.24 ^i^	9.04 ± 0.24
	Konini	nd	nd	nd	10.58 ± 0.69 ^ghi^	10.58 ± 0.69
30 min Fermenting	Öelands hvede	nd	2.44 ± 0.53 ^d^	1.31 ± 0.17 ^ab^	11.25 ± 0.67 ^egh^	15.01 ± 1.37
	Indigo	nd	nd	nd	10.57 ± 1.55 ^ghi^	10.57 ± 1.55
	Konini	nd	nd	nd	12.55 ± 1.13 ^ef^	12.55 ± 1.13
65 min Fermenting	Öelands hvede	4.19 ± 0.71 ^ef^	3.49 ± 0.09 ^c^	nd	13.34 ± 1.68 ^cde^	21.03 ± 2.49
	Indigo	nd	nd	nd	13.25 ± 2.06 ^de^	13.25 ± 2.06
	Konini	2.66 ± 0.37 ^f^	nd	nd	15.01 ± 0.69 ^bcd^	17.67 ± 0.44
Bread Loaf	Öelands hvede	11.07 ± 2.42 ^cd^	4.37 ± 0.29 ^dc^	1.51 ± 0.07 ^a^	13.53 ± 1.49 ^cde^	30.47 ± 4.27
	Indigo	7.39 ± 2.41 ^de^	5.20 ± 0.83 ^ab^	1.26 ± 0.06 ^b^	14.40 ± 0.42 ^bcd^	28.25 ± 3.66
	Konini	11.87 ± 1.93 ^bc^	3.44 ± 0.32 ^c^	1.15 ± 0.01 ^b^	15.53 ± 0.98 ^ab^	32.00 ± 2.53
Bread Crust	Öelands hvede	nd	3.73 ± 0.27 ^c^	nd	12.41 ± 0.90 ^ef^	16.15 ± 1.17
	Indigo	nd	5.19 ± 0.04 ^ab^	nd	12.01 ± 1.01 ^efg^	17.21 ± 1.05
	Konini	nd	6.00 ± 0.28 ^a^	0.70 ± 0.02 ^c^	15.11 ± 0.26 ^abc^	21.82 ± 0.55
Bread Crumb	Öelands hvede	16.29 ± 1.68 ^a^	nd	nd	14.76 ± 0.74 ^abcd^	31.05 ± 1.82
	Indigo	8.64 ± 1.87 ^cd^	nd	nd	14.65 ± 0.03 ^abcd^	23.29 ± 2.01
	Konini	15.24 ± 1.37 ^ab^	nd	nd	16.44 ± 0.57 ^a^	31.68 ± 1.54

Content of phenolic acid was expressed as μg/g of dry weight. Values in each column with different letters are significantly different (*p* < 0.05). Total phenolic acid was calculated as the sum of each row. ^a–^^j^ Significant difference was defined with different letters in each group. nd stands for not detected.

**Table 3:** Composition of insoluble-bound phenolic acids in normal and purple wheat grains at different stages of the bread-making process.

Steps	Sample Name	Protocatechuic Acid	*p-*Hydroxybenzoic Acid	Vanillic Acid	Caffeic Acid	Syringic Acid	*p-*Coumaric Acid	Ferulic Acid	Sinapic Acid	Total Phenolic Acid
Flour	Öelands hvede	nd	22.73 ± 1.29 ^b^	9.29 ± 0.54 ^f^	4.54 ± 0.18 ^k^	9.03 ± 0.58 ^b^	26.13 ± 1.19 ^a^	532.15 ± 30.00 ^defgh^	69.36 ± 2.69 ^cd^	673.25 ± 36.47
	Indigo	nd	25.69 ± 1.17 ^a^	24.82 ± 0.01 ^a^	6.53 ± 0.15 ^ef^	9.95 ± 0.23 ^a^	19.14 ± 1.50 ^def^	447.02 ± 24.14 ^kl^	35.97 ± 2.80 ^k^	569.12 ± 29.99
	Konini	nd	24.60 ± 1.24 ^a^	19.20 ± 0.97 ^b^	7.10 ± 0.30 ^bc^	9.05 ± 0.09 ^b^	21.64 ± 0.68 ^c^	466.66 ± 0.28 ^jk^	52.48 ± 2.24 ^hij^	600.74 ± 5.81
Mixing	Öelands hvede	20.67 ± 1.00 ^a^	10.63 ± 0.73 ^fg^	7.44 ± 0.49 ^h^	4.85 ± 0.02 ^jk^	9.46 ± 0.57 ^ab^	26.50 ± 0.39 ^a^	573.64 ± 11.84 ^bcd^	72.37 ± 0.88 ^bc^	725.56 ± 15.92
	Indigo	19.37 ± 0.60 ^ab^	24.48 ± 1.68 ^a^	9.48 ± 0.32 ^f^	4.52 ± 0.00 ^k^	5.97 ± 0.17 ^f^	19.63 ± 1.25 ^de^	482.89 ± 9.02 ^ijk^	51.74 ± 2.88 ^ij^	618.07 ± 15.94
	Konini	14.37 ± 0.84 ^e^	20.59 ± 1.19 ^c^	13.58 ± 0.18 ^de^	5.70 ± 0.28 ^g^	7.24 ± 0.36 ^de^	22.57 ± 1.16 ^bc^	514.29 ± 4.29 ^hi^	60.38 ± 0.28 ^ef^	658.73 ± 8.57
30 min	Öelands hvede	15.42 ± 0.43 ^d^	7.83 ± 0.39 ^h^	6.17 ± 0.17 ^h^	nd	nd	21.02 ± 0.96 ^cd^	411.57 ± 13.46 ^lm^	64.66 ± 2.88 ^de^	526.66 ± 18.23
	Indigo	15.46 ± 0.51 ^d^	11.63 ± 0.31 ^f^	8.68 ± 0.73 ^fg^	nd	nd	14.56 ± 0.54 ^ij^	363.99 ± 18.02 ^n^	40.82 ± 3.24 ^k^	455.14 ± 23.35
	Konini	11.13 ± 0.68 ^f^	13.79 ± 0.42 ^e^	12.76 ± 0.51 ^e^	nd	nd	13.57 ± 0.03 ^j^	386.39 ± 11.13 ^mn^	49.10 ± 2.84 ^j^	486.74 ± 15.60
65 min	Öelands hvede	17.56 ± 0.04 ^c^	11.70 ± 0.61 ^f^	12.86 ± 0.92 ^e^	5.20 ± 0.08 ^ij^	9.40 ± 0.46 ^ab^	26.57 ± 0.56 ^a^	526.64 ± 31.76 ^efghi^	71.83 ±1.05 ^bc^	681.75 ± 35.47
	Indigo	19.97 ± 0.23 ^ab^	12.19 ± 0.24 ^f^	19.18 ± 0.69 ^b^	6.21 ± 0.28 ^f^	6.83 ± 0.32 ^e^	16.90 ± 0.25 ^gh^	488.22 ± 2.63 ^hijk^	54.86 ± 0.48 ^ghi^	624.36 ± 5.12
	Konini	14.99 ± 0.22 ^de^	15.77 ± 0.91 ^d^	18.17 ± 0.98 ^b^	6.86 ± 0.05 ^cde^	7.82 ± 0.27 ^cd^	18.61 ± 0.19 ^efg^	523.25 ± 7.22 ^fghi^	60.34 ± 2.47 ^ef^	665.81 ± 12.31
Load	Öelands hvede	3.75 ± 0.12 ^ghi^	10.94 ± 0.27 ^fg^	14.68 ± 0.30 ^cd^	5.30 ± 0.22 ^hi^	9.63 ± 0.16 ^ab^	26.64 ± 0.30 ^a^	586.92 ± 30.32 ^bc^	75.51 ± 2.09 ^b^	733.38 ± 33.79
	Indigo	4.12 ± 0.08 ^gh^	9.99 ± 0.32 ^g^	19.32 ± 0.59 ^b^	6.62 ± 0.16 ^de^	6.97 ± 0.27 ^e^	17.31 ± 0.33 ^fgh^	517.14 ± 1.40 ^hi^	56.12 ± 1.48 ^fghi^	637.57 ± 5.16
	Konini	3.10 ± 0.08 ^i^	5.29 ± 0.15 ^ij^	18.65 ± 0.62 ^b^	7.33 ± 0.26 ^ab^	8.12 ± 0.09 ^c^	19.39 ± 1.47 ^de^	564.64 ± 37.89 ^cdef^	65.31 ± 1.81 ^de^	691.84 ± 43.37
Crust	Öelands hvede	nd	4.80 ± 0.21 ^ij^	15.45 ± 0.21 ^c^	5.66 ± 0.19 ^gh^	nd	27.87 ± 0.79 ^a^	631.54 ± 42.35 ^a^	80.92 ± 3.40 ^a^	766.23 ± 47.15
	Indigo	nd	4.44 ± 0.10 ^j^	19.49 ± 0.66 ^b^	7.17 ± 0.24 ^bc^	nd	19.30 ± 1.07 ^de^	551.35 ± 6.76 ^cdefg^	57.45 ± 4.15 ^fgh^	659.20 ± 12.98
	Konini	nd	4.25 ± 0.27 ^j^	18.77 ± 0.63 ^b^	7.63 ± 0.04 ^a^	nd	20.70 ± 4.12 ^cd^	616.95 ± 21.59 ^ab^	71.57 ± 3.64 ^bc^	739.88 ± 26.59
Crumb	Öelands hvede	3.95 ± 0.14 ^ghi^	6.17 ± 0.59 ^i^	14.51 ± 0.11 ^cd^	5.19 ± 0.05 ^ij^	9.80 ± 0.16 ^a^	23.93 ± 1.64 ^b^	569.32 ± 26.73 ^cde^	70.96 ± 4.66 ^bc^	703.82 ± 34.08
	Indigo	4.33 ± 0.17 ^g^	5.62 ± 0.21 ^ij^	18.33 ± 1.00 ^b^	6.56 ± 0.15 ^ef^	6.97 ± 0.38 ^e^	17.10 ± 0.63 ^gh^	498.80 ± 7.71 ^hij^	51.14 ± 0.89 ^ij^	608.85 ± 11.08
	Konini	3.25 ± 0.13 ^hi^	4.32 ± 0.27 ^j^	18.65 ± 1.21 ^b^	6.96 ± 1.64 ^bcd^	8.18 ± 0.13 ^c^	16.05 ± 0.98 ^hi^	554.64 ± 24.93 ^cdefg^	58.22 ± 0.79 ^fg^	670.28 ± 28.61

Content of phenolic acid was expressed as μg/g of dry weight. Values in each column with different letters are significantly different (*p* < 0.05). Total phenolic acid was calculated as the sum of each row. ^a–^^n^ Significant difference was defined with different letters in each group. nd stands for not detected.

### 2.4. Total Anthocyanin Content (TAC)

**Figure 4:**
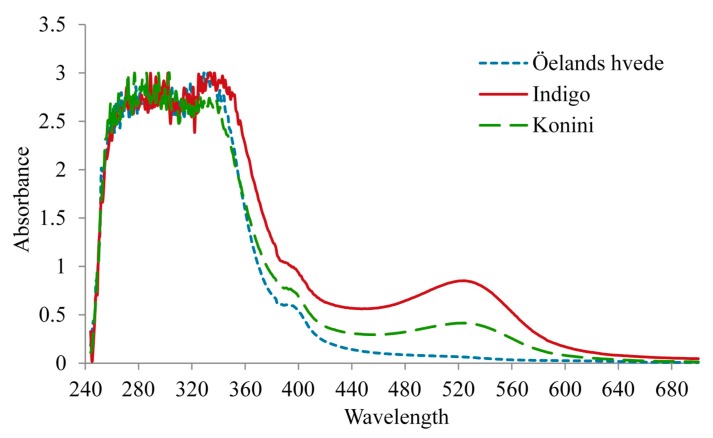
UV/Visible spectra of raw wheat flour at different wavelengths.

Spectra of absorbance at different wavelengths were identified for anthocyanin extract. As displayed in Figure 4, a wavelength range of 520 to 535 nm was detected to exert the highest absorbance for anthocyanins in Indigo and Konini varieties. No obvious peak was observed for Öelands hvede flour because of the low anthocyanin content. Giusti and Wrolstad [33] summarized the λ_(vis-max)_ for cyanidin-3-glucoside using different solvents, indicating the λ_(vis-max)_ of 510 nm for aqueous buffer, 512 nm for 10% ethanol, 520 nm for 0.1 N HCl, and 530 nm for 1% HCl in methanol. Therefore, wavelengths of 520 and 535 were used for detecting anthocyanins in acidified methanol extracts.

Indigo contained significantly (*p <* 0.05) higher anthocyanin content throughout the whole process of bread-making, ranging from 4.39 to 10.84 mg C3GE/100 g (Table 4). The lowest TAC was obtained from bread crust and the highest was from raw flour. The TAC values for Konini varied from 2.33 to 5.61 mg C3GE/100 g, lowest in bread crust and highest in raw flour. The non-colored wheat, Öelands hvede, contained the lowest TAC, which was approximately 8 and 4 times lower than the raw flours of Indigo and Konini, respectively.

With respect to the bread-making process, mixing significantly (*p* < 0.05) reduced TAC values. This was probably due to the dilution of anthocyanin content with other bread ingredients. Fermentation induced the release of anthocyanins and TAC gradually increased during fermentation. This effect was significantly (*p* < 0.05) seen at both the 30 min and 65 min fermenting steps. The TAC value increased up to 19% compared to the dough after mixing. Baking had a significant (*p* < 0.05) influence on anthocyanins, leading to a 54% to 55% decrease of TAC levels in the Konini and Indigo varieties, respectively. This result was in agreement with the study of Jing *et al.* [34], who stated that anthocyanins were relatively unstable and often underwent degradation during processing. Abdel-Aal and Hucl [35] investigated the stability of anthocyanins at different temperatures. They found that when the temperature increased from 65 °C to 95 °C, degradation also increased. Li *et al.* [8] reported a complete destruction of anthocyanins from purple wheat bran when heating processing (177 °C for 7–12 min) was used for muffin production. In the present study, the thermally labile property of anthocyanins was further confirmed by the TAC of bread fractions. Bread crust, which was exposed to the intense heat, contained the lowest TAC value. Bread crumb contained a relatively higher TAC value because the inner-side of bread possessed a less intense heat. In terms of the Öelands hvede variety, TAC was relatively stable throughout the whole process of bread-making. This indicated a minor level of anthocyanins in Öelands hvede.

**Table 4:** Total anthocyanin content in normal and purple wheat grains at different stages of the bread-making process.

Bread-Making Stages	Öelands hvede	Indigo	Konini
Flour *	1.45 ± 0.06 ^k^	10.84 ± 0.27 ^a^	5.61 ± 0.12 ^e^
Mixing *	1.06 ± 0.05 ^l^	8.50 ± 0.14 ^d^	4.38 ± 0.05 ^h^
30 min Fermenting *	1.35 ± 0.03 ^k^	8.95 ± 0.09 ^c^	5.05 ± 0.14 ^fg^
65 min Fermenting *	1.39 ± 0.03 ^l^	9.47 ± 0.31 ^b^	5.20 ± 0.04 ^f^
Bread Loaf *	1.02 ± 0.03 ^l^	4.85 ± 0.03 ^g^	2.60 ± 0.03 ^i^
Bread Crust *	0.86 ± 0.06 ^l^	4.39 ± 0.14 ^h^	2.33 ± 0.05 ^j^
Bread Crumb *	0.55 ± 0.02 ^m^	5.47 ± 0.03 ^e^	2.77 ± 0.06 ^i^

* Results were expressed as mg cyanidin-3-glucoside equivalents/100 g. Values with different letters are significantly different (*p <* 0.05).

### 2.5. Total Anthocyanin Content Using pH Differential Method

Table 5 summarized the total anthocyanin content using the pH differential method. Similar results were obtained using this method. TAC significantly (*p <* 0.05) decreased after mixing and gradually increased during fermenting. Baking destroyed anthocyanins and the exposure to intense heat led to the lowest TAC in bread crust. Compared to the result using direct measurement method, pH differential method detected lower TAC in Indigo and Konini extracts and detected no anthocyanins in Öelands hvede. This method is more accurate for determining TAC, since two aspects have been improved by the pH differential method: it (1) suppressed the interference of degraded anthocyanins and its derivatives; and (2) omitted the hazes or sediments.

**Table 5:** Total anthocyanin content using pH differential method.

Bread-Making Stages	Öelands hvede	Indigo	Konini
Flour *	nd	10.41 ± 0.39 ^a^	5.31 ± 0.02 ^d^
Mixing *	nd	8.09 ± 0.37 ^c^	4.22 ± 0.07 ^f^
30 min Fermenting *	nd	8.60 ± 0.47 ^bc^	4.65 ± 0.25 ^ef^
65 min Fermenting *	nd	8.97 ± 0.26 ^b^	4.80 ± 0.08 ^de^
Bread Loaf *	nd	4.64 ± 0.24 ^ef^	2.15 ± 0.04 ^g^
Bread Crust *	nd	4.16 ± 0.21 ^f^	1.99 ± 0.07 ^g^
Bread Crumb *	nd	4.81 ± 0.26 ^de^	2.18 ± 0.08 ^g^

* Results were expressed as mg cyanidin-3-glucoside equivalents/100 g. Values with different letters are significantly different (*p* < 0.05).

### 2.6. Antioxidant Activity of Anthocyanin Extracts

**Figure 5:**
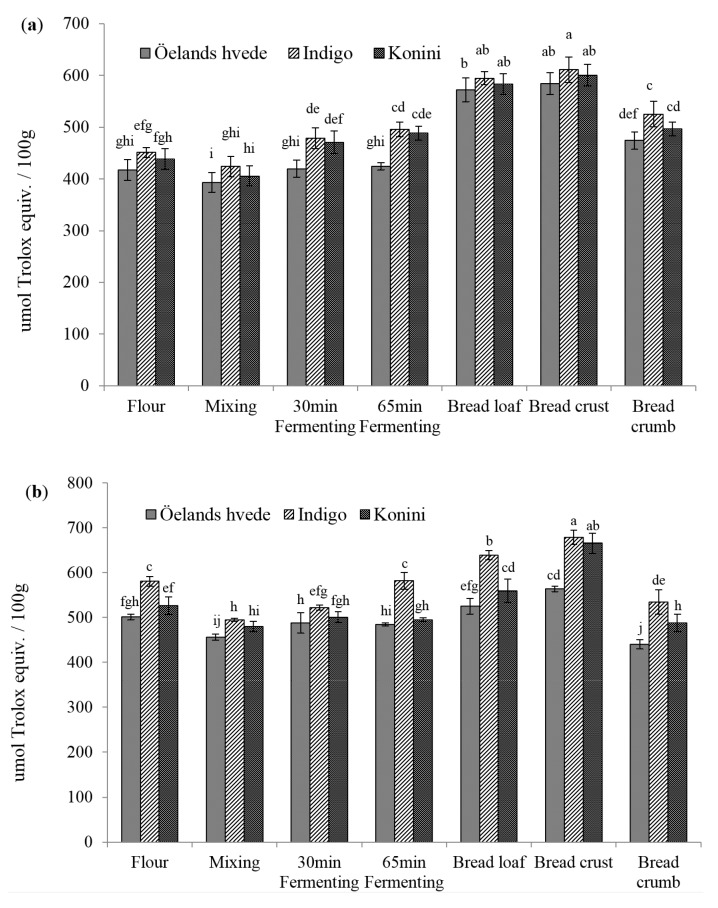
DPPH radical scavenging capacity (**a**) and ABTS radical cation decolorization activity (**b**) of anthocyanin extracts. Columns labeled with different letters are significantly different (*p* < 0.05).

Anthocyanin extracts during the production of bread were examined for their AOA against DPPH radical and ABTS radical cation (Figure 5). The DPPH values of purple wheat varieties were higher than that of normal wheat (Figure 5a). The significant (*p* < 0.05) difference was detected in fractions from the 30 min and 65 min fermenting steps. The high AOA of purple wheat can most likely be contributed to the presence of anthocyanins. Consistent to TAC, DPPH values decreased after mixing and gradually recovered during fermentation. Significant (*p* < 0.05) increases in AOA of 37%, 32%, and 33% for Öelands hvede, Indigo, and Konini, respectively, were observed in bread. Bread crusts exhibited the highest values. This could be explained by the extracting method, which not only targeted anthocyanins, but also extracted other soluble antioxidants such as phenolic acids and MRPs.

The effect of anthocyanins on AOA was better revealed by ABTS•^+^ decolorization activity assay (Figure 5b). Significant (*p* < 0.05) difference was detected between the purple and common wheat varieties. Indigo exhibited the highest ABTS values. The changes in ABTS levels during bread production were in accordance with the DPPH assay.

### 2.7. Analysis of Anthocyanin Composition

**Figure 6:**
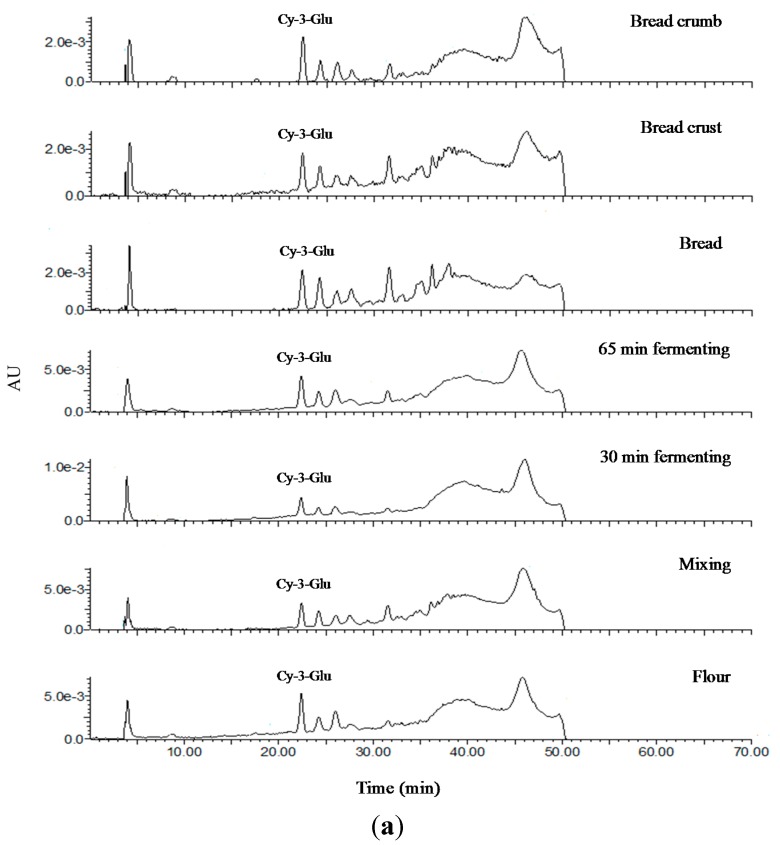

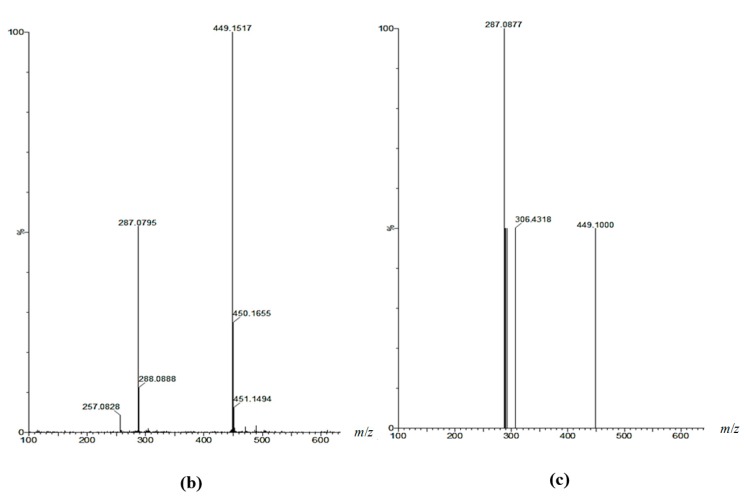
LC chromatogram (**a**) of anthocyanin at different stages of bread-making as well as LC-MS (**b**) and LC-MS/MS of cyanidin-3-glucoside (cy-3-glu) (**c**) in flour.

To examine the presence of anthocyanins, an HPLC chromatogram with MS and MS/MS was recorded at a wavelength of 520 nm. Anthocyanin composition detected by HPLC-QTOF-MS/MS is shown in Figure 6. Only one main peak was recognized as anthocyanin using MS/MS, namely cyanidin-3-glucoside (RT = 22.48 min). Two ions were evident for the identification including *m*/*z* 499 ([M + H]^+^), being the molecular weight of anthocyanin, and fragment ion *m*/*z* 287 arising from a loss of glucose residue *m*/*z* 162. This was in agreement with the study of Hosseinian *et al.* [36]. Liu *et al.* [5] also detected cyanidin-3-glucoside in Indigo and Konini wheat without the presence of other anthocyanins.

## 3. Experimental Section

### 3.1. Chemicals and Standards

Analytical grade acetic acid, Folin-Ciocalteu reagent, 2,2′-diphenyl-1-picrylhydrazyl (DPPH), 2,2′-azinobis-(3-ethylbenzothiazoline-6-sulfonic acid) (ABTS), 6-hydroxy-2,5,7,8-tetramethychroman-2-carboxylic acid (Trolox), and phenolic acid standards (gallic, protocatechuic, *p-*hydroxybenzoic, vanillic, caffeic, syringic, *p-*coumaric, ferulic, and sinapic acid) were purchased from Sigma-Aldrich Chemical Co. (St. Louis, MO, USA). HPLC grade methanol and ethyl acetate were purchased from Fisher Scientific Co. (Ottawa, ON, Canada). Deionized water (Milli-Q) was used in the HPLC analysis.

### 3.2. Sample Description

**Figure 7:**
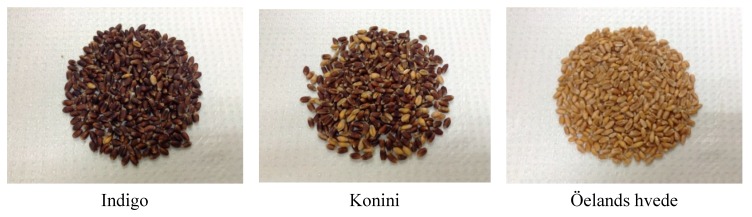
Photographs of whole wheat grains.

Three bread wheat grains (*Triticum aestivum*) were received from Mørdrupvej 5 DK-3540 Lynge (Lynge, Denmark), including Indigo (97% purple kernels), Konini (84% purple kernels), and Öelands hvede (yellow kernels). These three wheat grains (Figure 7) were all spring wheats grown in 2012 at Mørdrupård, 30 km northeastern from Copenhagen, Denmark from a certified organic farm (latitude N 55°49.530′, longitude E 012°13.587′). The grains were milled using a Häusler Mühle model HS with a 50 cm Naxos stone (Häussler, Karl-Heinz Häussler GmbH, Albstadt, Germany).

### 3.3. Bread-Making Procedure

Bread was baked according to the Canadian short process method (CSP method, Canadian Grain Commission) [37] with some modifications. The formula included 100 g flour (14% moisture basis), 3 g compressed yeast, 2.5 g gluten, 4 g whey powder, 4 g sugar, 2.4 g salt, 1 g malt, and 3 g pure lard. The farinograph absorption ((FAB) was determined by a Brabander DoCorder model 2200-3 (Brabender Instruments, Inc., South Hackensack, NJ, USA) which was modified by the Grain Research Lab (GRL, Winnipeg, MB, Canada). The amount of liquid added was calculated as: (FAB-3 mL) water (37 °C) + 1 mL ammonium phosphate solution (0.1%). Mixing took place in a pin mixing bowl (100 g Mixer, National Manufacture Co., Lincoln, NE, USA) at 140 rpm and 30 °C. Optimum mixing time was 10% past peak consistency. The dough was fermented in a warm cabinet (12 door-proofer with humidity and temperature controller) at 37.5 °C and 85% relative humidity. After 15 min, the dough was punched lightly seven times by hand. Afterwards, the dough was placed back into the warm proofing cabinet for another 15 min and subsequently was processed through a sheeter (GRL manufactured sheeter, Canadian Grain Commission) at 11/32 and 3/16 levels, respectively. The dough was molded immediately using a GRL molder for 30 s. The bread dough was proved for 35 min, which was determined by maximum height. The bread baking oven (Model 6 Precision Scientific, National Manufacturing Company, Lincoln, NE, USA) was preheated to a set temperature of 200 °C. The dough was baked for 25 min. To avoid the introduction of antioxidants, pure lard was used instead of shortening. Each sample was produced in two batches and the average value was reported.

### 3.4. Sample Preparation

At the end of mixing, 30 min fermenting, 65 min fermenting, and baking, samples were removed immediately into a −20 °C freezer to terminate the chemical changes. All the samples were freeze-dried along with the raw flour, bread crust (5 mm thick outside the whole bread), and crumb (crust removed bread inner-side) and ground to pass through a 0.42 mm sieve. All the samples were stored at 4 °C before analysis.

### 3.5. Extraction of Soluble Phenolic Compounds

A finely ground sample (2 g) was extracted twice with 80% methanol at a ratio of 1:5 (*w*/*v*) (total: 20 mL of 80% methanol). Each time, the mixture was shaken for 1.5 h using a wrist action shaker (Burrell, Pittsburgh, PA, USA) and then sonicated for 0.5 h (Branson 5510, Richmond, VA, USA) under nitrogen and dark condition at ambient temperature. The mixture was centrifuged at 11,963 *g* and 4 °C for 15 min (Sorvall RC 6+, Thermo Fisher Scientific, Ottawa, ON, Canada). The supernatants were combined and filtered with a φ 90 mm filter paper (Whatman™ Cat No. 1004-125). The collected crude extracts were stored at −20 °C until the analysis for free phenolic content, DPPH• scavenging activity, and ABTS•^+^ decolorization capacity. To identify and quantify the soluble phenolic acids, the crude extracts were filtered with 0.45-μm syringe filters (VWR International, Cat No. 28146-489) prior to the injection into HPLC. The residues were dried in a fume hood at room temperature and kept in sealed bags at 4 °C before alkaline hydrolysis. Extraction was done in duplicate.

### 3.6. Extraction of Insoluble Phenolic Compounds

The residue collected after 80% methanol extraction was subjected to alkaline hydrolysis. Insoluble phenolic compounds were released using the method described by Qiu *et al.* [27] with some modifications. First, 15 mL of 4M NaOH was added to the dried residue (0.5 g) and hydrolyzed on a shaking water bath (VWR, Radnor, PA, USA) for 4 h under N_2_ and at 250 rpm and 25 °C. The hydrolyzed mixture was adjusted to a pH between 1.5 and 2.0 with 6 N HCl and then extracted three times with ethyl acetate (25 mL ×3). Every time after mixing, ethyl acetate was separated from the aqueous layer by centrifuging at 11,963 *g* and 4 °C for 5 min (Sorvall RC 6+, Thermo Fisher Scientific, Ottawa, ON, Canada). The supernatant was collected into an Erlenmeyer flask using a pipette. Further dehydration was performed by adding 1 g of Na_2_SO_4_ and then filtering with a filter paper (φ 90 mm, Whatman™ Cat No. 1004-125). The combined ethyl acetate extracts were evaporated and dried at 35 °C under a vacuum using a rotary evaporator (Yamato RE-51, Cole-Parmer Instrument Company, Bunker Court Vernon Hill, IL, USA) with a water bath (Thermo-Lift, Fisher Scientific, Missouri, NJ, USA). The dried residue was redissolved in 2 mL of 50% methanol and stored at −20 °C. The extract was used for the determination of bound phenolic content, DPPH• scavenging activity, and ABTS•^+^ decolorization capacity. To investigate the presence of insoluble phenolic acids, HPLC analysis was also performed. The samples were filtered with 0.45-μm syringe filters (VWR International, Cat No. 28146-489) before injection. Extraction was done in duplicate.

### 3.7. Extraction of Anthocyanins

Extraction of anthocyanins was performed according to a method summarized by Young and Abdel-Aal [38] with modifications. Specifically, a finely ground sample (1 g) was extracted with acidified methanol/HCl (1 N) (85:15, *v*/*v*) at a ratio of 1:8 (*w*/*v*). The pH of the mixture was adjusted to 1. The mixture was shaken for 1.5 h (Wrist action shaker, Burrell, Pittsburgh, PA, USA), followed by sonication for 0.5 h (Branson 5510, Richmond, VA, USA) under nitrogen and in the dark at ambient temperature. The mixture was then centrifuged at 11,963 *g* and 4 °C for 15 min (Sorvall RC 6+, Thermo Fisher Scientific, Ottawa, ON, Canada). The supernatant was filtered with a φ90 mm filter paper (Whatman™ Cat No. 1004-125) and evaporated to dryness at 35 °C under a vacuum using a rotary evaporator (Yamato RE-51, Cole-Parmer Instrument Company, Bunker Court Vernon Hill, IL, USA) with a water bath (Thermo-Lift, Fisher Scientific, Missouri, NJ, USA). The residue was then redissolved in 2 mL of 80% methanol. The crude extracts were kept at −20 °C for two days to precipitate large molecules and then filtered through a 0.45-μm syringe filter (VWR International, Cat No. 28146-489). The extracts were stored at −20 °C prior to the determination of total anthocyanin content (TAC) with pH differential method, DPPH• scavenging activity, and ABTS•^+^ decolorization capacity. Extraction was done in duplicate.

### 3.8. Determination of Phenolic Content

The free, bound, and total phenolic contents (FPC, BPC, and TPC) were determined using a Folin-Ciocalteau method as described by Li *et al.* [8]. Briefly, 10-fold dilution of a Folin-Ciocalteu reagent was prepared just prior to use. Then 1.5 mL of freshly diluted Folin–Ciocalteau reagent was used to oxidize 0.2 mL sample extracts. After allowing the mixture to equilibrate for 5 min, the reaction was then neutralized with 1.5 mL sodium carbonate solution (60 g/L) at room temperature. The absorbance of the resulting solution was measured at 725 nm after 90 min against a blank of 80% or 50% methanol (80% methanol for soluble phenolic extracts and anthocyanin extracts, 50% methanol for insoluble phenolic extracts). Ferulic acid (FA) and gallic acid (GA) were used as standards. Therefore, the free, bound, and total phenolic content of samples were expressed as mg of FAE (equivalents)/100 g and mg of GAE/100 g.

### 3.9. DPPH Radical Scavenging Capacity Activity Assay

The DPPH method was used according to the modified method used by Beta *et al.* [4]. A 60 μmol/L DPPH• reactant was made in methanol. Then 3.9 mL of DPPH• solution was added to 0.1 mL of sample and the absorbance at 515 nm was measured at *t* = 30 min. To determine the absorbance at *t* = 0 min, measurement was taken by adding 3.9 mL of DPPH• solution to 0.1 mL of 80% or 50% methanol (80% methanol for soluble phenolic extracts and anthocyanin extracts, 50% methanol for insoluble phenolic extracts). The antioxidant activity was calculated as: % DPPH• scavenging activity = (1 − [*A*_sample,*t=*30_/*A*_control,*t=*0_]) × 100. A plot of trolox concentration *vs.* % DPPH• scavenging activity was used as a standard curve. DPPH value was expressed as μmol TE (Trolox equivalents)/100 g.

### 3.10. ABTS Radical Cation Decolorization Assay

The ABTS•^+^ reagent was made by mixing ABTS•^+^ stock solution (7 mM) (38.4 mg ABTS in 10 mL distilled water) and potassium persulfate stock solution (2.45 mM) (6.62 mg potassium persulfate in 10 mL distilled water) at a ratio of 1:1 for 2 min vortex. The mixture was stored for 12 to 16 h in the dark at room temperature prior to use. Then, 5 mL of ABTS•^+^ reagent was diluted with approximately 495 mL of distilled water and absorbance at a wavelength of 734 nm was adjusted to 0.7 by diluting the solution further with distilled water. The antioxidant activity of soluble or insoluble phenolic extracts and anthocyanin extracts (50 μL) was evaluated by adding 1.85 mL of freshly made ABTS•^+^ reagent. The absorbance was determined at *t* = 30 min. For absorbance at *t* = 0 min, 1.85 mL of freshly made ABTS•^+^ reagent was added to 50 μL of 80% or 50% methanol (80% methanol for soluble phenolic extracts and anthocyanin extracts, 50% methanol for insoluble phenolic extracts). The ABTS•^+^ decolorization (%) was calculated as (1 − [*A*_sample, *t*=30_/*A*_control,*t*=0_]) × 100. A standard curve was generated based on different trolox concentrations *vs.* % ABTS•^+^ decolorization and the antioxidant activities of extracts were expressed as μmol TE/100g sample.

### 3.11. Absorbance Spectra of Anthocyanin Extract at Different Wavelengths

A finely ground sample (1 g) was extracted with acidified methanol/HCl (1 N) (85:15, *v*/*v*) at a ratio of 1:8 (*w*/*v*). The mixture was shaken for 0.5 h (Wrist action shaker, Burrell, Pittsburgh, PA, USA) under nitrogen and dark condition at ambient temperature. The mixture was then centrifuged at 11,963 *g* and 4 °C for 15 min (Sorvall RC 6+, Thermo Fisher Scientific, Ottawa, ON, Canada). The supernatant was filtered with a φ90 mm filter paper (Whatman™ Cat No. 1004 125). The absorbance of the collected supernatant was scanned at wavelengths from 230 to 700 nm using an Ultrospec 1100 Pro UV/Visible spectrophotometer (Amersham Biosciences, Centreville, VA, USA) controlled by Biochrom Data Capture Software (version 2.0) (Biochrom Ltd., Cambridge, UK). A plot of absorbance *vs.* wavelength was created.

### 3.12. Direct Measurement of Total Anthocyanin Content

Total anthocyanin content was measured according to the method reported by Liu *et al.* [5] with some modifications. Briefly, 8 mL of acidified methanol/HCl (1 N) (85:15, *v*/*v*) with pH = 1 was added to 1 g of finely ground sample. The mixture was shaken for 1.5 h (Wrist action shaker, Burrell, Pittsburgh, PA, USA), followed by sonication for 0.5 h (Branson 5510, Richmond, VA, USA) under nitrogen and in the dark at ambient temperature. The mixture was centrifuged at 11,963 *g* and 4 °C for 15 min (Sorvall RC 6+, Thermo Fisher Scientific, Ottawa, ON, Canada). The supernatant was filtered with a φ 90 mm filter paper (Whatman™ Cat No. 1004-125). The absorbance at 535 nm was measured without further dilution of the crude extracts. Cyanidin-3-glucoside was used as a standard, which was the primary anthocyanin in wheat (Abdel-Aal *et al.*, 2006). The total anthocyanin content of each sample was calculated as:

(1) $$\frac{\text{A }\times \text{ Vol }\times \text{MW }\times \;10^{2}\;}{\text{ε }\times \text{ Sample Wt}}$$

where A is absorbance reading; MW is molecular weight of cyanidin-3-glucoside (449.2 g/mol); ε is molar absorptivity of cyanidin-3-glucoside (25965/cm/M); and Wt is dry weight of the ground grain sample. The results were expressed as mg of C3GE (cyanidin-3-glucoside equivalents)/100 g sample.

### 3.13. Total Anthocyanin Content using the pH Differential Method

A second procedure for determination of total anthocyanin content is based on a pH differential method [39]. Theoretically, anthocyanin pigments undergo reversibly structural transformations when pH changes. This leads to a color change, which can be monitored by spectrophotometer (colored at pH = 1.0, colorless at pH = 4.5). To conduct this assay, two buffer solutions were prepared: one for pH = 1.0 potassium chloride buffer (0.03 M) and the other for pH = 4.5 sodium acetate buffer (0.4 M). Briefly, 1.9 g of KCl was dissolved into 980 mL distilled water and the pH was adjusted to 1 with 6 N HCl, meanwhile, 54.4 g CH_3_CO_2_Na∙3H_2_O was dissolved in 960 mL distilled water and the pH was adjusted to 4.5 with acetic acid. Anthocyanin extracts that had been reconstituted into 2 mL of 80% methanol were used in this assay. Samples were diluted five times with both buffers to a final volume of 2 mL, respectively (0.4 mL of anthocyanin extracts in 1.6 mL of either potassium chloride buffer or sodium acetate buffer). The absorbance of each sample was measured at 520 nm against a blank of distilled water after 30 min of preparation. The haze and sediment were corrected by measuring the absorbance again at 700 nm. The concentration (mg/L) of each anthocyanin was calculated according to the following formula and expressed as mg C3GE/100 g sample:

(2) $$\frac{\text{A }\times \text{ MW }\times \text{ DF }\times \;10^{3}\;}{\text{ε}\times \;1}$$

where A is the absorbance = (A_520nm_ − A_700nm_) _pH 1.0_ − (A_520nm_ − A_700nm_) _pH 4.5_, MW is the molecular weight for cyanidin-3-glucoside = 449.2 g/mol, DF is the dilution factor = 5, L is the path length = 1 cm, and ε is the extinction coefficient = 26,900 L × cm^−1^ × mol^−1^ for cyanidin-3-glucoside.

### 3.14. Identification and Quantification of Phenolic Compounds with High Performance Liquid Chromatographu

Identification and quantification of phenolic compounds were carried out on an HPLC (Waters 2695, Milford, MA, USA) equipped with a photodiode array (PDA) detector (Waters 996, Milford, MA, USA) and an autosampler (Waters 717 plus, Milford, MA, USA). Analysis was performed on a Gemini 5 μ C18 110 A column (150 mm × 4.60 mm) (Phenomenex, Torrance, CA, USA) and the mobile phase consisted of 0.1% acetic acid in water (solvent A) and 0.1% acetic acid in methanol (solvent B). A 70 min linear gradient was programmed as follows: 0–11 min, 9%–14% B; 11–14 min, 14%–15% B; 14–17 min, 15% B; 17–24 min, 15%–16.5% B; 24–28 min, 16.5%–19% B; 28–30 min, 19%–25% B; 30–36 min, 25%–26% B; 36–38 min, 26%–28% B; 38–41 min, 28%–35% B; 41–46 min, 35%–40% B; 46–48 min, 40%–48% B; 48–53 min, 48%–53% B; 53–65 min, 53%–70% B; 65–66 min, 70%–9% B; 66–70 min, 9% B. The injection volume was 10 μL and the flow rate was 0.9 mL/min. Identification of phenolic acids was achieved by comparison to the retention time of phenolic acid standards and their maximum UV absorption. A sample spiked with external standards was used for further identification. Phenolic acid quantitation was based on the standard curves of the corresponding phenolic acids at a wavelength of 280 nm and peak area was used for calculations. The HPLC analyses were done in duplicate for each sample.

### 3.15. Identification of Anthocyanins in Purple Wheat with HPLC-QTOP-MS/MS

The anthocyanin composition was evaluated following a method described by Bicudo *et al.* [40]. The chromatographic separation was carried out using a HPLC (Waters 2695) system equipped with photodiode array detector (Waters 996) and autosampler (Waters 717 plus) (Waters Corporation, Milford, MA, USA). A Gemini 5 μm RP-18 (150 mm × 4.6 mm) (Phenomenex, Torrance, CA, USA) analytical column was used for separation. Each anthocyanin extract was injected at a volume of 10 μL for analysis. The mobile phase consisted of (A) water with 0.1% formic acid and (B) methanol with 0.1% formic acid at a flow rate of 0.5 mL/min. A linear gradient was programmed as follows: 0–5 min, 5%–10% B; 5–15 min, 10%–15% B; 15–20 min, 15%–20% B; 20–30 min, 20%–25% B; 30–40 min, 25%–40% B; 40–45 min, 40%–10% B; 45–50 min, 10% B. Anthocyanins were detected at a wavelength of 280 nm. The quadrupole time-of-flight mass spectrometer (Q-TOF-MS) (Micromass, Waters Corp., Milford, MA, USA) was calibrated by using sodium iodide for the positive mode through the mass range of 100–1500. MS parameters were set as follows: capillary voltage: 2100 V, sample cone voltage: 30 V, source temperature: 120 °C, desolvation temperature: 250 °C, desolvation gas (nitrogen gas) flow rate: 900 L/h. The MS/MS spectra were acquired by using a collision energy of 30 V.

### 3.16. Statistical Analysis

The results were reported as mean ± standard deviation (SD) of duplicate determinations. Analysis of variance (ANOVA) for main factors (flour type, process step, and flour type* process step) was determined using the GLM procedure with SAS version 9.3 (SAS Institute Inc., Cary, NC, USA). Least significant differences (LSD) were employed to define significant differences among varieties at a level of *p* < 0.05. Pearson’s correlation test was used to evaluate the correlation among variables at significant levels of *p* < 0.05 and *p* < 0.01.

## 4. Conclusions

In summary, the changes of FPC, BPC, TAC as well as their AOA during the production of bread were first systematically investigated. Individual phenolic acids were also identified and quantified in fractions from each stage of bread-making. Mixing, fermenting, and baking significantly increased FPC and BPC. Bread crust contained the highest FPC, while bread crumb contained the highest BPC. AOA were correlated to their respective phenolic content. Different phenolic acids behaved differently in the bread-making process, which might be ascribed to their difference in chemical natures, sensitivities towards heat, and ease of liberation from plant cell walls. TAC was significantly reduced through mixing and baking, but fermentation elevated the level of TAC. Anthocyanin extract of purple wheat exerted higher AOA than the common wheat, indicating the potential of purple wheat to be used as a functional ingredient in baking.
